# Dietary intake assessment in children with cystic fibrosis using 3-day food diaries: a single-centre study

**DOI:** 10.3389/fped.2023.1130792

**Published:** 2023-05-09

**Authors:** Margaux Gaschignard, Fabien Beaufils, Pauline Gallet, Haude Clouzeau, Joris Menard, Aurélie Costanzo, Lucie Nouard, Laurence Delhaes, Candice Tetard, Thierry Lamireau, Michael Fayon, Stéphanie Bui, Raphaël Enaud

**Affiliations:** ^1^ Pediatric Cystic Fibrosis Reference Center (CRCM), Bordeaux University Hospital, Hôpital Pellegrin-Enfants, Centre d’Investigation Clinique (CIC 1401), Bordeaux, France; ^2^ Centre de Recherche Cardio-Thoracique de Bordeaux, Bordeaux University, INSERM U1045, U1045, Bordeaux, France

**Keywords:** cystic fibrosis, nutrition, nutritional status, nutritional intake, macronutrient, micronutrient

## Abstract

**Background:**

Malnutrition is both a feature and major cause of morbidity in cystic fibrosis (CF). Therefore, nutritional management is an essential element of patient care. In 2016, an international guideline for nutritional management in patients with CF was published. In light of these recommendations, the aim of this study was to investigate the dietary intake of children with CF at the University Hospital of Bordeaux.

**Methods:**

We conducted a retrospective study at the Paediatric CF Centre of the University Hospital of Bordeaux. Patients aged 2–18 years with CF who completed a 3-day food diary at home between January 2015 and December 2020 were included.

**Results:**

A total of 130 patients, with a median age of 11.8 [interquartile range (IQR): 8.3; 13.4] years, were included. The median Z-score for BMI was −0.35 (IQR: −0.9; 0.2) and 20% of the patients had a *Z*-score for BMI < −1. Recommended total energy intakes were achieved in 53% of the patients, particularly those with nutritional support. Recommended protein intake was met in 28% of the cases, while fat and carbohydrate intakes were met in 54%. Vitamin and micronutrient levels were normal in 80% of the patients, with the exception of vitamin K, which was within the therapeutic range in only 42% of the cases.

**Conclusion:**

Recommended nutritional targets are difficult to achieve in patients with CF, and providing nutritional support during follow-up remains a challenge.

## Introduction

Cystic fibrosis (CF), which is the predominant genetic disease in European populations, is caused by mutations in the gene encoding the cystic fibrosis transmembrane conductance regulator (CFTR) protein. The resulting ion transport defect leads to multiple organ dysfunction, where respiratory dysfunction is the most common prognostic determinant (1).

Since its initial description by Dorothy Andersen in 1938, malnutrition has been recognised as a major cause of morbidity in CF. Several factors contribute to malnutrition in CF, including low energy intake, high energy expenditure, exocrine pancreatic insufficiency (EPI), chronic lung inflammation and infection, intestinal inflammation, increased essential fatty acid turnover, high salt loss in sweat and impaired bicarbonate secretion (2–4). Therefore, nutritional support is an essential component of CF management (5, 6).

Neonatal screening and multidisciplinary follow-up for CF, which were implemented in France in 2002, have improved the nutritional management of patients (7). Furthermore, the 2016 joint international recommendations of the European Society of Clinical Nutrition and Metabolism (ESPEN), European Society of Paediatric Gastroenterology, Hepatology and Nutrition (ESPGHAN), and European Cystic Fibrosis Society (ECFS) have also emphasized the principles of nutritional management for patients with CF. In particular, a high-calorie diet (accounting for 110%–200% of the basic energy requirement) should be followed (8). Energy intake should be adjusted according to age, sex, physical activity, and disease status to obtain satisfactory growth and optimal nutritional status, and to limit disease progression (9).

The present study investigated the dietary intake of CF children at the University Hospital of Bordeaux in light of the ESPEN-ESPGHAN-ECFS recommendations.

## Methods

### Study design and population

This retrospective study was conducted at the University Hospital of Bordeaux, and included children with CF (sweat test > 60 mmol/L) aged 2–18 years, who completed a 3-day food diary between January 2015 and December 2020 to assess the dietary intake at home. Patients were included regardless of their nutritional status, related comorbidities (e.g., EPI, diabetes, or liver disease), and treatment (e.g., nutritional support). Only one 3-day food diary was analysed for each patient, with the most recent one being preferred. The 3-day food diaries were completed prior to CFTR modulator therapy if required.

Data were extracted exclusively from the medical records of the patients using MUCODOMEOS software® (version 3.7.1) (10) after obtaining informed consent. In accordance with French laws, ethics committee approval was not required for this study.

### Three-day food diary

In addition to systematic evaluations by the dieticians of the CF unit during follow-up, dietary intake assessment included a 3-day food diary, kept as part of the routine care of children with CF at the University Hospital of Bordeaux. The diaries were kept by all patients within 3 months of their annual check-up regardless of nutritional status. The diaries were maintained at home by stable patients (no exacerbation for at least 3 months). Due to the workload of the CF team dietitians, the food diaries were maintained with assistance from a dietitian from a home care provider, and therefore the diaries were only available to patients who already had a home care provider (mainly patients on nebuliser therapy or enteral nutrition).

The 3-day food diary consisted of a detailed records of all daily meals and snacks, as well as nutritional supplements and medications, for 3 consecutive days (including 1 weekday and 2 weekend days). Parents recorded the quantities of food consumed according to weight or household units. The protein, fat, carbohydrate, micronutrient and vitamin contents of the foods were calculated according to the CIQUAL 2012 food composition table (https://ciqual.anses.fr). The food diaries were then sent to the dieticians of the CF unit for analysis and comparison with the recommendations.

Macronutrient intake was expressed as grams per kilogram per day (g/kg/d), kilocalories per kilogram per day (kcal/kg/day), and a percentage of the recommended total energy intake (TEI). TEI was the sum of spontaneous food intake and any nutritional support, including oral nutritional supplements (ONS) and EN.

Compliance with the recommendations was defined as a TEI of 110%–200% of the recommended dietary intake for the general population according to age and sex, a moderate level of physical activity, and a diet consisting of 45% carbohydrates, 35% fats and 20% protein (8). The following recommended nutritional intakes were applied to CF patients:

- TEI > 100 kcal/kg/day (110EN%) for patients aged 2–6 years [recommended value: 90 kcal/kg/day for the general population in this age group (11)], including at least 5 g/kg/day of protein (20EN%), 3.9 g/kg/day of fats (35EN%), and 11.3 g/kg/day of carbohydrates (45EN%).
- TEI > 83 kcal/kg/day (110EN%) for patients aged 7–11 years [recommended value: 75 kcal/kg/day (11)], including at least 4.2 g/kg/day of protein (20EN%), 3.2 g/kg/day of fats (35EN%), and 9.3 g/kg/day of carbohydrates (45EN%).
- TEI > 67 kcal/kg/day (110EN%) for patients aged ≥ 12 [recommended value: 60 kcal/kg/day (11)], including at least 3.3 g/kg/day of protein (20EN%), 2.6 g/kg/day of fats (35EN%), and 7.5 g/kg/day of carbohydrates (45EN%).

### Clinical and biological data

Demographic and CF-related data were collected from the patients’ medical records, including age, sex, CFTR gene mutations, comorbidities (EPI, meconium ileus, liver disease, glucose intolerance and diabetes, and chronic *Staphylococcus aureus* or *Pseudomonas aeruginosa* colonisation), treatments (particularly vitamin supplementation), anthropometric data (weight, height, and body mass index [BMI], expressed as *Z*-scores calculated from French references (12)), respiratory function [percentage of predicted forced expiratory volume in 1 s (FEV1pp)], and blood vitamin and micronutrient levels.

Clinical and biological data were obtained during annual routine care visits. Chronic *P. aeruginosa* colonisation was defined as the presence of *P. aeruginosa* isolates in three consecutive cultures, with at least 1 month between positive cultures, during the previous 6 months (13).

### Statistical analysis

The results were expressed as means ± standard deviation (SD) or medians and interquartile range (IQR). Categorical variables are expressed as absolute values and percentages. The data were analysed using R software® (version 4.0; R Foundation, Vienna, Austria). The data were compared among age groups and according to the presence of nutritional support. Normality was assessed using the Shapiro-Wilk test. Parametric variables were compared using analysis of variance or Student's t-test, while non-parametric variables were compared using the Kruskal-Wallis or Mann-Whitney test. Categorical variables were analysed using Fisher's exact test or the Chi-square test. Correlations were assessed using Pearson's or Spearman's correlation coefficient. Missing data were managed using the complete case analysis method. A *p*-value < 0.05 was considered significant.

## Results

**Figure 1:**
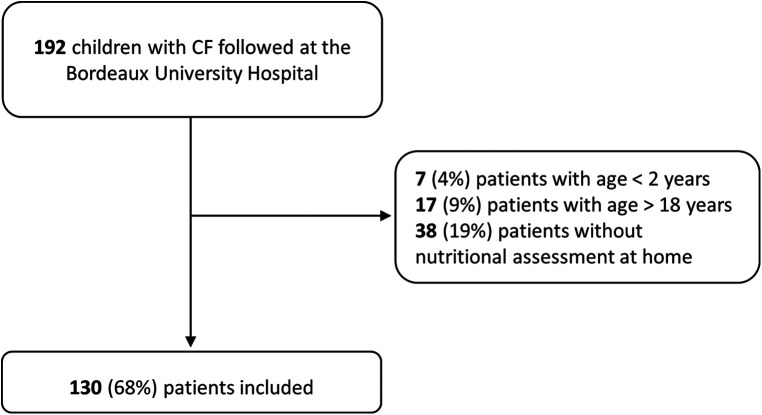
Flow chart.

Of the 192 children with CF seen at the University Hospital of Bordeaux, 130 were included in this study; 9 3-day food diaries were completed in 2015, 15 in 2016, 11 in 2017, 14 in 2018, 47 in 2019, and 34 in 2020. In total, 62 (32%) patients were excluded; 38 (19%) did not have a 3-day food diary, and 24 (13%) were aged > 18 years or <2 years at the time of the 3-day food diary (Figure 1).

Patient characteristics are summarised in Table 1. The median age of the patients was 11.8 (IQR: 8.3; 13.4) years and 62 (48%) were female. Furthermore, 68 (52%) patients were F508del homozygous, while 51 (39%) were F508del heterozygous. The median FEV1pp was 84% (IQR: 73; 102). Chronic *S. aureus* and *P. aeruginosa* colonisation were present in 72% and 21% of the patients, respectively, and 84% of the patients had EPI.

**Table 1:** Patient characteristics.

	All patients (*n* = 130)	2 to 6 years old (*n* = 20)	7 to 11 years old (*n* = 55)	12 to 18 years old (*n* = 55)	*p* *
Female gender	62 (48%)	12 (60%)	29 (53%)	21 (38%)	0.61
Age (years)	11.8 [8.3; 13.4]	5 [2.9; 5.7]	9.9 [8.5; 11.1]	13.8 [12.9; 16.2]	**<0**.**01**
CFTR mutations					
F508del homozygous	68 (52%)	10 (50%)	30 (55%)	28 (51%)	0.20
F508del heterozygous	51 (39%)	7 (35%)	21 (38%)	23 (42%)	
Others	11 (9%)	3 (15%)	4 (7%)	4 (7%)	
FEV1pp	84 [73; 102]	89 [70; 110]	86 [76; 105]	83 [66; 99]	0.4
Chronic *Staphylococcus aureus* colonisation	93 (72%)	10 (50%)	36 (65%)	47 (86%)	**0**.**02**
Chronic *Pseudomonas* a*eruginosa* colonisation	27 (21%)	3 (15%)	6 (11%)	18 (33%)	**<0**.**01**
*Z*-Score Weight	−0.1 (±1.1)	0.2 (±1.3)	0.1 (±1.2)	−0.4 (±1.0)	**0**.**03**
*Z*-Score Height	0.2 (±1.2)	0.9 (±1.2)	0.4 (±1.1)	−0.2 (±1.2)	**<0**.**01**
*Z*-Score BMI	−0.3 (±1.0)	−0.7 (±0.9)	−0.1 (±1.0)	−0.4 (±0.9)	**0**.**01**
*Z*-Score < 0	84 (65%)	13 (65%)	34 (62%)	37 (67%)	0.88
*Z*-Score < −1	26 (20%)	5 (25%)	9 (16%)	12 (22%)	0.61
Nutritional support					
ONS	32 (25%)	2 (10%)	13 (24%)	17 (31%)	0.12
EN	15 (12%)	1 (5%)	8 (15%)	6 (11%)	0.57
EPI	109 (84%)	19 (95%)	41 (75%)	49 (89%)	**0**.**03**
Pancreatic enzymes (U/kg/day)	6,395 [4,637; 76,301]	7,692 [6,911; 8,858]	6,528 [4,756; 7,631]	5,660 [4,540; 7,267]	**0**.**02**
Pancreatic enzymes (U/g of fat/day)	1,725 [1,459; 2,576]	1,631 [1,454; 2,146]	1,676 [1,212; 2,603]	1,822 [1,537; 2,739]	0.32
Glucose intolerance	43 (33%)	0	15 (27%)	28 (51%)	**<0**.**01**
Diabetes	18 (14%)	1 (5%)	4 (7%)	13 (24%)	**0**.**03**
Liver disease	23 (18%)	2 (10%)	9 (16%)	12 (22%)	0.69
Meconium ileus	16 (12%)	2 (10%)	8 (15%)	6 (11%)	0.60

Data are expressed as *n* (%), mean (±SD) or median [IQR].

CFTR, cystic fibrosis transmembrane conductance regulator; EN, enteral nutrition; EPI, exocrine pancreatic insufficiency; ONS, oral nutritional supplements; FEV1pp, percentage of predicted forced expiratory volume in 1 s.

Significant values (*p* < 0.05) are in boldface.

* For the comparison between the three age groups.

The mean Z-scores for weight, height, and BMI were −0.1 ± 1.1, 0.2 ± 1.2, and −0.3 ± 1.0, respectively. Eighty-four (65%) patients had a *Z*-score < 0, while twenty-six (20%) had a *Z*-score < −1 for BMI. There was a positive correlation between BMI and FEV1pp (*r *= 0.39*; p* < 0.001).

Macronutrient intakes are summarised in Table 2. The median TEI was 80 (IQR: 62; 105) kcal/kg/day. Sixty-nine children met the recommended TEI, representing 53% of the sample. Patients with and without EPI showed no significant difference in TEI (82 [IQR: 64; 108] and 69 [IQR: 59; 89], respectively; *p* = 0.2). TEI was negatively correlated with FEV1pp (*r* = −0.3; *p* < 0.01) and BMI (*r* = −0.42; *p* < 0.001).

**Table 2:** Macronutrients intakes.

	All patients (*n* = 130)		2 to 6 years old (*n* = 20)		7 to 11 years old (*n* = 55)		12 to 18 years old (*n* = 55)		*p* *
	Median intake	Complies with recommendations^a^	Median intake	Complies with recommendations^a^	Median intake	Complies with recommendations^a^	Median intake	Complies with recommendations^a^	
TEI (kcal/kg/d)	80 [62; 105]	69 (53%)	114 [85; 124]	12 (60%)	82 [64; 98]	31 (56%)	66 [45; 91]	26 (47%)	0.39
Food consumption (kcal/kg/d)	73 [58; 90]	55 (42%)	113 [85; 120]	12 (60%)	73 [63; 86]	23 (42%)	64 [45; 79]	20 (36%)	0.37
Nutritional support (kcal/kg/d)	14 [9; 30]	—	41 [23; 49]	—	17 [9; 41]	—	12 [9; 25]	—	—
Total Carbohydrate intake (g/kg/d)	9.5 [6.7; 12.2]	70 (54%)	12.2 [10.2; 14.9]	11 (55%)	9.6 [7; 11.6]	30 (55%)	7.8 [5.4; 11.6]	29 (53%)	0.91
Total Protein intake (g/kg/d)	3.1 [2.3; 4]	36 (28%)	4 [3.2; 4.5]	3 (15%)	3.1 [2.5; 4]	13 (24%)	2.6 [1.9; 3.6]	20 (36%)	0.16
Total fat intake (g/kg/d)	3.1 [2.4; 4.5]	70 (54%)	4.8 [3.6; 5.4]	14 (70%)	3.3 [2.5; 4.5]	28 (51%)	2.6 [1.8; 3.4]	28 (51%)	0.2

Data are expressed as *n* (%) or median [IQR].

TEI, Total energy intake.

^a^ Number (%) of patients whose intakes were consistent with recommendations. .

* Comparison of compliance with recommendations between the three age groups.

The median carbohydrate intake was 9.5 (IQR: 6.7; 12.2) g/kg/day, corresponding to 47% (IQR: 43; 50) of the TEI; 70 children (54%) met the carbohydrate intake recommendations. The median protein intake was 3.1 (IQR: 2.3; 4) g/kg/day, representing 16% (IQR: 14; 17) of the TEI. Thirty-six children met the protein intake recommendations, representing 28% of the cohort. The median fat intake was 3.1 (IQR: 2.4; 4.5) g/kg/day, representing 36% (IQR: 34; 40) of the TEI. Seventy children met the fat intake recommendations, representing 54% of the cohort.

The vitamin and micronutrient supplementation and blood level data of our cohort are shown in Tables 3 and 4, respectively*.* Vitamin A and D supplementation was provided to nearly all the patients. Vitamin E and zinc supplementation was given to 78% of the cohort, while vitamin K and zinc supplementation was given to about two-thirds of the patients. Vitamin and micronutrient levels were within the therapeutic range in about 80% of the cases; the only exception was vitamin K, which was within the therapeutic range in only 42% of the cases.

**Table 3:** Vitamin and micronutrient supplementation in our cohort (*n* = 130).

	Recommended substitution	Substituted patients	Dosage	Prescription according to the recommendations^a^
Vitamin A (U/d)	To be adapted according to biological standards	126 (97%)	1,667 [1,250; 11,558]	–
Vitamin E (U/d)	100 à 400 UI/d	101 (78%)	329 [291; 736]	79 (78%)
Vitamin D (U/d)	800 UI/d (up to 2,000 UI/d)	129 (99%)	1,441 [870; 3,480]	99 (77%)
Vitamin K (mg/month)	1 to 10 mg/d	93 (72%)	40 [10; 40]	81 (87%)
Selenium (µg/d)	To be adapted according to biological standards	82 (63%)	100 [100; 100]	–
Zinc (mg/d)	15 mg/d	101 (78%)	5 [5; 6.25]	17 (17%)

Data are expressed as *n* (%) or median [IQR].

^a^ The recommended values are taken from the ESPEN-ESPGHAN-ECFS dietary recommendations published in 2016. These recommendations have been adapted for patients with exocrine pancreatic insufficiency. It should be noted that there is currently no specific recommendation for selenium replacement. Zinc supplementation is not systematic, but is recommended for those at risk of deficiency, particularly in the presence of delayed puberty or growth or anorexia.

**Table 4:** Dosages of vitamins and micronutrients.

	All patients (*n* = 130)		2 to 6 years old (*n* = 20)			7 to 11 years old (*n* = 55)			12 to 18 years old (*n* = 55)			*p* *
	Median level	Within the laboratory standards	Laboratory standards	Median level	Within the laboratory standards	Laboratory standards	Median level	Within the laboratory standards	Laboratory standards	Median level	Within the laboratory standards	
Vitamin A (µmol/l)	1.2 [1.0; 1.5]	101 (78%)	*0.7–1.5*	1.2 [1.0; 1.4]	11 (55%)	*0.9–1.7*	1.2 [1.0; 1.3]	41 (75%)	*0.9–2.5*	1.3 [1.1; 1.6]	49 (89%)	**0**.**006**
Vitamin E (µmol/l)	21 [17; 25]	106 (82%)	*7–21*	18 [10; 27]	11 (55%)	*14–23*	23 [19; 25]	53 (96%)	*12–42*	19 [16; 24]	42 (76%)	**<0**.**001**
Vitamin K (mg/l)	176 [80; 325]	55 (42%)	*150–900*	261 [56; 473]	7 (35%)	*150–900*	223 [99; 329]	18 (33%)	*150–900*	117 [73; 229]	30 (55%)	**0**.**05**
Vitamin D (ng/ml)	37 [30; 46]	120 (92%)	*10–55*	41 [32; 41.5]	10 (50%)	*10–55*	40 [30; 47]	55 (100%)	*10–55*	35 [30; 44]	55 (100%)	**<0**.**001**
Zinc (µmol/l)	14.0 [13; 15]	110 (85%)	*9–22*	13.6 [12; 15.5]	15 (79%)	*9–22*	14 [13; 15]	42 (76%)	*9–22*	14 [12; 16]	53 (96%)	**0**.**002**
Selenium (µmol/l)	1.0 [0.9; 1.2]	108 (83%)	*0.75–1.5*	1.2 [1; 1.31]	16 (80%)	*0.75–1.5*	1.0 [0.9; 1.2]	43 (78%)	*0.75–1.5*	1.0 [0.9; 1.9]	49 (89%)	0.31

Data are expressed as *n* (%) or median [IQR].

Significant values (*p* < 0.05) are in boldface.

* Comparison between the 3 age groups of the rate of dosages within the laboratory standards.

Forty-seven (36%) patients received nutritional support, with ONS and EN being provided to 32 (25%) and 15 (12%) patients, respectively. Compared to the patients without nutritional support, those receiving nutritional support were older (12.0 ± 3.2 years *vs*. 10.6 ± 4.3 years; *p* = 0.04), had worse lung function (mean FEV1pp: 75 ± 21 *vs*. 92 ± 20; *p* < 0.001), and had lower BMI Z-scores (−0.6 ± 0.7 *vs.* −0.1 ± 1.0; *p* < 0.01). The Z-scores for weight and height were also lower in patients with nutritional support (−0.5 ± 1.0 *vs.* 0.1 ± 1.2; *p* < 0.01 and −0.1 ± 1.3 *vs.* 0.4 ± 1.2; *p* < 0.03, respectively). TEI was higher in patients with nutritional support (96 [IQR: 77; 115] kcal/kg/d *vs.* 67 [IQR: 52; 90]; *p* = 0.001), whereas food-related energy intakes were identical in both groups (76 [IQR: 66; 89] kcal/kg/d and 67 [IQR: 52; 90] kcal/kg/d for patients with and without nutritional support, respectively, *p* = 0.6). Recommended intakes were met more often in patients with nutritional support compared to those without nutritional support, including TEI (81% *vs*. 38%; *p* < 0.001), carbohydrate intake (77% *vs*. 42%; *p* < 0.001), protein intake (55% *vs*. 12%; *p* < 0.001), and fat intake (79% *vs*. 41%; *p* < 0.001).

Age was negatively correlated with the *Z*-scores for weight and height (*r* = −0.3; *p* < 0.01 and *r* = −0.4; *p* < 0.001, respectively), but not with those for BMI. However, the Z-score for BMI was lower in the 2–6-years age group (Table 1). There was no significant correlation between age and FEV1pp. There was also no difference in the likelihood of meeting the TEI, carbohydrate, protein or fat intake recommendations among age groups (Table 2).

## Discussion

This single-centre study investigated the dietary intakes of children with CF at the University Hospital of Bordeaux and compared them with the ESPEN-ESPGHAN-ECFS recommendations. In our cohort of 130 children, the mean *Z*-score for BMI was −0.3 (± 1.0), while the median *Z*-score in the ECSF Patient Registry 2020 was −0.2 (IQR: −0.9; 0.5) (14). The majority of the patients (65%) had a BMI *Z*-score < 0, while 20% had a BMI *Z*-score < −1. In addition, age was negatively correlated with the *Z*-scores for weight and height. An increased risk of malnutrition in CF has been reported previously (15, 16). According to the French CF Registry 2020 (*n* = 7376), 53% of the females and 59% the males aged 0–18 years had BMI *Z*-scores < 0 (17). We also found a positive correlation between BMI and FEV1pp. The associations of poor nutritional status with decreased lung function and increased mortality are well-established in patients with CF (18, 19). Conversely, good nutritional status is associated with improved lung function and survival (15, 18, 20, 21).

Malnutrition in patients with CF is multifactorial, involving increased energy loss (malabsorption) and expenditure (especially in relation to inflammation and chronic infections) (15). TEI did not differ significantly between our patients with and without EPI, but was negatively correlated with FEV1pp and BMI, indicating that severe CF patients have increased energy expenditure and, therefore, greater energy needs. Poulimeneas et al. demonstrated that patients with a BMI < 50th percentile had higher TEIs compared to those with BMI ≥ 50th percentile (22). Malnutrition in patients with CF may also be related to inadequate dietary intake (15). Therefore, it was necessary to assess dietary intake in children with CF. Despite regular multidisciplinary management, including dietary consultations at each follow-up visit, only 53% of the children in our study met the ESPEN-ESPGHAN-ECFS TEI recommendations for patients with CF (8). In a 2017 study of 75 preschool CF children with EPI by Filigno et al*.* approximately 45% of the children achieved 110% of the recommended food intake (23). In another study conducted in six European CF centres, the rate was as high as 60% (24).

Current recommendations suggest that dietary intake should consist of 20% proteins, 35%–40% fats, and 40%–45% carbohydrates (8). In our cohort, the macronutrients distribution was 25%, 33% and 42% from proteins, lipids, and carbohydrates, respectively, compared to 13%, 35%, and 52% in Filigno et al. (23)*.* Similar distributions were reported in a European multicentre study by Calvo-Lerma et al. (24). However, the recommended carbohydrate and fat intakes were achieved in about half of the patients, whereas protein intake was achieved in only a quarter. A high-calorie and -fat diet is recommended for patients with CF, along with adequate pancreatic enzyme supplementation for those with EPI; this may explain these results (8, 15, 19). A recent systematic review concluded that there was no association between macronutrient distribution and nutrition-related outcomes, and questioned the indication for a high-fat diet, which is only beneficial for obtaining more energy from lower food volumes (16). In addition, the ESPEN-ESPGHAN-ECFS recommendations, which are based on recent studies, suggest that protein intake should be emphasized to maintain muscle mass and improve the long-term prognosis (8, 20).

One third of our patients received nutritional support: ONS in 32 (25%) patients and EN in 15 (12%) patients. Patients with nutritional support had poorer lung and nutritional statuses than those without nutritional support. The frequency of nutritional support in our cohort was similar to that reported in the French CF Registry 2020 (ONS and EN in 30% and 6% of the children aged 0–18 years, respectively) (17). Recommended TEI and macronutrient intakes were achieved twice as often with nutritional support. Sufficient protein intake was achieved in 55% of the cases with nutritional support compared to 12% of those without nutritional support. This may be because the solutions used are mostly high in protein. A Cochrane review of three randomised clinical trials concluded that there was no effect of ONS on nutritional status, including BMI, in moderately malnourished children with CF after 12 months of use (25). However, the nutritional benefits of supplementation were demonstrated by Shepherd et al*.* and Rettammel et al*.* (26, 27). The benefits of EN are less controversial. A recent study demonstrated beneficial effects of EN on weight, height, growth velocity, and BMI in patients with CF aged > 5 years (28). Although our study could not conclusively demonstrate a beneficial effect of nutritional support on the nutritional status of children with CF, the results support the use of nutritional supplementation in patients with poor nutritional status despite increased dietary intake and pancreatic enzyme replacement therapy.

Vitamin and micronutrient levels were within the therapeutic range in about 80% of our cases. The only exception was vitamin K, which was within the therapeutic range in only 42% of the cases. This may be because of poor compliance with vitamin K due to its taste, which may improve with the recent development of multivitamin and water-soluble formulations. It should also be noted that, contrary to expectations, blood levels were generally better in the 12–18 years age group compared to the other age groups.

This study had several limitations. In our centre, due to the workload of the CF team dietitians, the three-day food diaries were maintained with the assistance of a home care dietitian and therefore the diaries were only available to patients who already had a home care provider. This may have led to selection bias, particularly for children aged < 6 years, because homecare providers are not systematically used before this age except in the most severe cases that require nutritional supplementation or early nebulization. This may explain why the *Z*-Scores for BMI were lower in the 2–6 years age group. In addition, the 3-day food diaries were questionnaires completed at home by the patients and their parents, which may have biased the data collection. In addition, the food quantities consumed were approximated in some cases because the food was not weighed systematically. We analysed only one 3-day food diary for each patient, even though food intake in CF children may exhibit considerable day-to-day variation depending on health status, appetite, and the level of physical activity. Repeated assessments are required to account for intra-individual variability. In addition, dietary records do not adequately assess micronutrient intake (e.g., sodium intake), which may be relevant in children with CF. Other factors, such as essential fatty acid intake, should also be considered as they may play a role in the growth and prognosis of patients with CF (29).

A final limitation of this study was that the dietary assessments were performed in the absence of CFTR modulators, which are used in the majority of patients. Improvements in nutritional status have been demonstrated in several studies with different combinations of CFTR modulators (30–33). The improvement is likely to be multifactorial, including improved absorption due to reduced intestinal inflammation, increased intestinal pH, and even improved pancreatic exocrine function (34–36). Improved lung function and reduced exacerbations due to modulators may also reduce energy expenditure and thus improve nutritional status. Against this background, it is necessary to evaluate changes in dietary intake in response to treatment and update the recommendations accordingly.

## Conclusions

This cross-sectional study highlights the continuing challenge of meeting nutritional targets in children with CF, despite established recommendations and multidisciplinary care. Although our results showed that approximately half of the children did not meet the nutritional targets, further research is needed to confirm the effectiveness of nutritional support through ONS or EN. Nevertheless, this study highlights the importance of continuing efforts to improve the nutritional care of children with CF.

## Data Availability

The raw data supporting the conclusions of this article will be made available by the authors, without undue reservation.
